# Chronic Illness and Quality of Life 5 Years After Displacement Among Rohingya Refugees in Bangladesh

**DOI:** 10.1001/jamanetworkopen.2024.33809

**Published:** 2024-09-17

**Authors:** Ahmed Hossain, Redwan Bin Abdul Baten, Altaf Saadi, Juwel Rana, Taifur Rahman, Hasan Mahmud Reza, Mohamad Alameddine

**Affiliations:** 1 College of Health Sciences, University of Sharjah, Sharjah, United Arab Emirates; 2 Department of Public Health, North South University, Dhaka, Bangladesh; 3 Department of Public Health Sciences, University of North Carolina, Charlotte; 4 Massachusetts General Hospital, Harvard Medical School, Boston; 5 Department of Epidemiology, Biostatistics and Occupational Health, McGill University, Montreal, Quebec, Canada; 6 Department of Public Health, Missouri State University, Springfield

## Abstract

**Importance:**

Rohingya refugees, forcibly displaced from Myanmar, face challenges adapting to Bangladesh. Examining their quality of life (QOL) is vital to identifying nuanced factors associated with their well-being, informing targeted interventions for an improved QOL.

**Objectives:**

To identify the QOL among Rohingya refugees 5 years after migration to Bangladesh, with a particular emphasis on understanding the complex interplay between sociodemographic factors and chronic illnesses.

**Design, Setting, and Participants:**

A cross-sectional study involving resettled Rohingya adults was conducted between May 18 and July 7, 2021, approximately 5 years after their resettlement in Bangladesh. Of the participants, 500 individuals were healthy, whereas 558 individuals were undergoing treatment for at least 1 chronic disease. Data were analyzed from January to February 2024.

**Main Outcomes and Measures:**

The study assessed QOL using the short version of the World Health Organization’s QOL Questionnaire, covering 4 domains: physical, psychological, social, and environmental. Scores were transformed to a maximum of 100. Tobit linear regression, adjusted for potential confounders, was employed for analysis.

**Results:**

The study included a total of 1058 respondents, who were predominantly female (630 participants [59.5%]) and had a mean (SD) age of 42.5 (16.1) years. Despite being healthy, individuals without chronic illnesses had median QOL scores ranging from 44 to 56 out of 100, indicating a relatively poor QOL. A total of 260 participants (46.6%) with chronic diseases reported very poor or poor QOL, in contrast to 58 healthy individuals (11.6%) in the fifth year after displacement. Specifically, patients with cancer and those who had multimorbidity exhibited the lowest QOL scores across all domains, with significant reductions in the physical health (10.57 decrease; 95% CI, −12.97 to −8.17) and psychological domain scores (7.20 decrease; 95% CI, −9.71 to −5.93) according to Tobit regression analysis.

**Conclusions and Relevance:**

This study found that chronic illnesses were associated with all domains of QOL among Rohingya refugees, particularly those with musculoskeletal disorders, cancer, and multimorbid conditions. This heightened vulnerability may contribute to poor QOL in this population. By uncovering these disparities, the study lays the groundwork for targeted interventions and policies aligned with the United Nations’ goal of leaving no one behind in sustainable development efforts.

## Key Points

**Question:**

What chronic illnesses are most prevalent among Rohingya refugees and how are they associated with quality of life (QOL)?

**Findings:**

This cross-sectional study of 1058 Rohingya refugees revealed a high prevalence of poor QOL, even after 5 years of displacement. Individuals without chronic illnesses exhibited the highest QOL scores, but their median scores indicated a relatively low QOL; in contrast, 46.6% of participants with chronic diseases reported very poor or poor QOL compared with 11.6% of healthy individuals.

**Meaning:**

These findings suggest individuals with chronic conditions like musculoskeletal disorders, cancer, and multimorbidity exhibit significantly lower well-being than healthier counterparts.

## Teaser

This cross-sectional study evaluates quality of life among Rohingya refugees 5 years after migration to Bangladesh, particularly among those with chronic illness.

## Introduction

There are over 110 million forcibly displaced people worldwide, meaning that 1 in 74 people are forcibly displaced.^1^ Refugees are a subset of the forcibly displaced population, representing people who are fleeing persecution for reasons of race, religion, nationality, or membership of a particular social group or political opinion, and whose claim for international protection has been granted. While there are common elements of trauma and persecution among all refugees, they still represent a heterogenous group with widely differing experiences, backgrounds, health needs, and health behaviors, which existing studies rarely acknowledge.^2,3^ This current study centers on refugees who have undergone resettlement in Bangladesh from Myanmar.

The Rohingya refugees make up one of the world’s largest refugee populations.^2^ A Muslim ethnic minority, Rohingya have experienced decades of persecution in Myanmar’s Rakhine state due to their religion and ethnic identity.^4^ More than 742 000 Rohingya people became refugees when they entered Bangladesh in August 2017 due to a massive wave of violence.^5^ That number has currently risen to nearly 1 million refugees living in Cox’s Bazar district, Bangladesh, an area known as the world’s largest refugee camp.^1^ Refugees, including the Rohingya, experience diverse health challenges, including a higher prevalence of diseases. Contributing factors include interruptions of care and lack of access to care.^6,7,8,9,10,11^

Chronic diseases, known as noncommunicable diseases (NCDs), pose a significant threat to Rohingya refugees in Cox’s Bazar due to limited access to care, crowded living conditions, poor mental health, unhealthy behaviors, and existing inequalities.^12,13,14,15,16,17,18,19^ Understanding the NCDs and sociodemographic factors influencing their quality of life (QOL) is crucial to inform a multifaceted approach that can deliver tangible improvements in their overall health and well-being.

This study investigated the QOL of Rohingya refugees 5 years after resettlement in Bangladesh, considering various well-being domains. Previous research indicates that posttraumatic stress symptoms persist among Rohingya refugees,^3^ while another study shows that asylum seekers have a lower QOL compared with healthy refugees.^20^ However, little is known about the QOL of Rohingya refugees and how chronic conditions impact their overall QOL. This study aimed to address this gap by comparing the QOL of Rohingya refugees with and without chronic diseases and identifying associated factors.

## Methods

### Study Design and Participants

The cross-sectional study was conducted between May 18 and July 7, 2021, in Cox’s Bazar and Ukhia Health Camp, which are camps established by the Government of Bangladesh, supported by the United Nations (UN) and other nongovernmental organizations. Thus, this study was conducted approximately 5 years after the refugees’ resettlement in Bangladesh. The study encompassed 1058 Rohingya refugees residing in the camps in Cox’s Bazar. Prospective participants were recruited from the largest refugee camp, Kutupalong, using a stratified sampling technique, consisting of 2 strata: individuals with chronic diseases and healthy individuals. Initially, individuals with chronic diseases were conveniently selected from 2 Health Management in Broader Dimension (HMBD) and 3 Young Power in Social Action (YPSA) medical centers. Both HMBD and YPSA are nongovernmental organizations working for the well-being of Rohingya refugees and host communities in Bangladesh. The study recruited a total of 558 individuals diagnosed with chronic diseases from various medical centers. These chronic conditions were identified and diagnosed by physicians who visited the medical centers and assessed the participants. Another 500 healthy individuals (without chronic diseases) were chosen from the households of Kutupalong, with the study targeting an adult individual from each household. The sample allocation of individuals is given in eTable 1 and eTable 2 in in Supplement 1. Within each household, 1 respondent, preferably the head of the household without a chronic disease, was interviewed. If the head of the household was unavailable, the following head or another adult member was surveyed. Household members were defined as individuals living together under the same roof for at least a month while sharing cooking and eating facilities from the same source. Additionally, participants were required to have resided in the camp for a minimum of 2 years after displacement.

The research protocol was approved by the North South University institutional review board and ethics review committee. The Refugee Relief and Repatriation commissioner issued the permission for data collection on May 4, 2021. The participants were informed that participation in the study was voluntary and provided written consent or verbal assent. Face-to-face interviews were conducted individually to maintain privacy. No financial or food incentives were provided. Participants were made aware that there was no incentive for participation or penalties in case of refusal to participate. The interview questions were read aloud, allowing the respondents to select their preferred response. The data collection sheets were reviewed for completeness and accuracy by coinvestigators and confirmed by the principal investigator. To guarantee data quality, coinvestigators reviewed all sheets for completeness, accuracy, and consistency. This review process was then double checked by the principal investigator. This study adhered to the reporting guidelines outlined in Strengthening the Reporting of Observational Studies in Epidemiology (STROBE).

### Recruitment and Training

The HMBD Foundation, a local NGO operating in the Rohingya camps, collaborated with YPSA medical centers to collect data from patients with diseases. These centers were informed about the research study and its ethical considerations. The HMBD Foundation, with the assistance of the local leaders (ie, majis from the camp), recruited 4 local data collectors who were proficient in both Bangla and Rohingya languages. A data collection team was formed, pairing a member from the camp and another from the HMBD Foundation for collecting data from healthy individuals. The interviews were conducted in both Rohingya and Bangla. The camp-based data collectors asked questions in Rohingya, and the HMBD members verified the responses by asking the same questions in Bangla.

Two research investigators (A.H. and T.R.) from North South University organized a 1-day practical training session on ethics and data collection. Enumerators were briefed on study objectives, methods, and questionnaire content. They were trained in report building, maintaining neutrality, ethical issues, privacy concerns, cultural awareness, and risk management for chronic conditions. Following the training, a pilot study was conducted by the 4 study teams to evaluate their comprehension of the techniques and troubleshoot any issues encountered during interviews. Necessary corrections were made based on the pilot study’s outcomes. Subsequently, each trained team visited their assigned camp to collect data using the semistructured questionnaire.

### Sociodemographic Factors and Disease Status

The questionnaire’s introductory section gathered sociodemographic information, including age (in years), sex (male or female), education, marital status (never married, widowed or divorced, and married), and primary occupation. Education was divided into 2 groups: those who had attended school or madrasa and those who did not go to school or madrasa. Additionally, participants diagnosed with chronic health conditions such as hypertension, diabetes, heart diseases, chronic kidney diseases, cancer or tumors, musculoskeletal disorders, or multimorbidity by the medical centers were categorized as the patient group. Multimorbidity is defined as the coexistence of 2 or more chronic conditions in an individual. The individuals who did not have any chronic conditions or any symptoms of chronic condition were considered the healthy group.

### Outcome Variable: QOL

The WHOQOL-BREF is a 26-item self-report questionnaire designed by the World Health Organization (WHO) to evaluate an individual’s QOL.^21,22^ The first 2 questions of WHOQOL-BREF presents the overall QOL and overall health status. The questionnaire comprises 4 main domains: physical health (7 items), encompassing inquiries about pain, energy levels, sleep, and mobility; psychological health (6 items), covering aspects of mood, self-esteem, and positive feelings; social relationships (3 items), addressing inquiries about support from others and satisfaction with relationships; and environment (8 items), incorporating questions related to the physical environment, safety, financial resources, and opportunities for leisure.

Each individual item of the WHOQOL-BREF is scored from 1 to 5 on a response scale, which is stipulated as a 5-point ordinal scale. The scores are then transformed linearly to a 0 to 100 scale with higher scores indicating better QOL.^21^ Providing a score out of 100 allows for easier interpretation and comparison between domains and populations. The WHOQOL-BREF has been translated into over 100 languages and is used in various settings, including clinical trials, research studies, and clinical practice.^21^ The WHOQOL-BREF is a reliable and valid measure of QOL. It has been used to assess QOL in various populations, including people with chronic diseases, mental health conditions, and disabilities.^22,23^ The WHOQOL-BREF can track QOL changes over time, compare QOL between different groups, identify areas where QOL interventions may be needed, and is a comprehensive tool to understand QOL holistically.

### Statistical Analysis

We used R version 4.3.1 (R Project for Statistical Analysis) for analysis. The first 2 questions of WHOQOL-BREF, concerning overall QOL and overall health status, were evaluated separately. Statistical analyses employing Wilcoxon tests (for 2 categories) or Kruskal-Wallis tests (for multiple categories) were employed to explore the potential relationships between the different domains of QOL and a range of sociodemographic factors. These tests were used to assess if there were any statistically significant differences in quality-of-life measures across different sociodemographic groups. We used the Tobit regression model because the WHO-QOL score is discrete, and some of the responses were missing, which were treated as censored. The Tobit model is a regression model used to estimate linear associations between variables when there are missing responses in the dependent variable. The Tobit model is used when the dependent variable is treated as censored at a known threshold and estimated using maximum likelihood estimation. Since the QOL score is bounded (which cannot be below 7 for physical health domain), Tobit regression can provide more meaningful and less biased results compared with linear regression. The tobit function from the AER package was used to estimate the slope (β) of the exposures.^24^ To test whether the slope coefficient (β) was significantly different from 0, a 2-sided statistical test was performed at a 5% significance level. The percentage of missing data in the covariates is presented in eTable 3 in Supplement 1. Since the missing information was less than 1% for the majority of covariates, we chose not to perform any data imputation. The questionnaire, R scripts, and data are available online.^25^ Data were analyzed from January to February 2024.

## Results

### Response Rate

Of the 564 patients sampled, 6 were excluded because they either did not consent to participate, or they were too ill to give an interview. Additionally, among the 519 healthy participants from households, 19 were excluded due to lack of consent or no eligible individuals being found in the houses during the study period. Consequently, our analysis included 1058 participants, resulting in a response rate of 97.8%. Further details on the response rate calculation can be found in the eAppendix in Supplement 1.

### Characteristics of the Participants

Table 1 presents the characteristics of the participants, offering insights into the demographics of adults with and without chronic illnesses. The sample comprised 1058 participants, with a higher proportion of female participants (630 individuals [59.5%]). There was a greater prevalence of chronic illnesses among female participants. The mean (SD) age of participants was 42.5 (16.1) years. As anticipated, individuals with chronic illnesses were predominantly in the older population (60 years and above). Regarding education, 934 participants (88.3%) had no formal schooling, while the remaining 124 (11.7%) had some level of education. Most participants were married (864 individuals [81.7%]), with a smaller percentage being unmarried (75 participants [7.1%]) or divorced/widowed (124 participants [11.2%]). However, the divorced or widowed individuals were mostly among those with chronic illnesses. In terms of occupation, nearly half of the participants were housewives (518 participants [49.4%]), while some were employed part-time (253 participants [24.1%]), and others were not working (277 participants [26.4%]). Housewives and participants who were not working were predominantly among those with chronic illnesses.

**Table 1.** Characteristics of the Respondents

Characteristic	Total (N = 1058)	Without chronic disease (n = 499 [47.2%])	With chronic disease (n = 558 [52.8%])
Sex			
Male	428 (40.5)	221 (51.6)	207 (48.4)
Female	630 (59.5)	278 (44.2)	351 (55.8)
Age, y			
Mean (SD)	42.5 (16.1)	NA	NA
18-24	149 (14.1)	132 (88.6)	17 (11.4)
25-34	226 (21.4)	176 (77.9)	50 (22.1)
35-44	170 (16.1)	97 (57.1)	73 (42.9)
45-54	241 (22.8)	58(24.2)	182 (75.8)
55-64	166 (15.7)	28 (16.9)	138 (83.1)
≥65	106 (10)	8 (7.5)	98 (92.5)
Schooling			
Had schooling	124 (11.7)	89 (71.8)	35 (28.2)
Never had schooling	934 (88.3)	410 (43.9)	523 (56.1)
Marital status			
Married	864 (81.7)	422 (48.9)	441 (51.1)
Unmarried	75 (7.1)	58 (77.3)	17 (22.7)
Divorced/widowed	119 (11.2)	19 (16.0)	100 (84.0)
Employment status			
Housewives	518 (49.4)	217 (41.9)	301 (58.1)
Paid work in the last month	253 (24.1)	157 (62.9)	96 (37.9)
Not working in the last month	277 (26.4)	125 (45.1)	152(54.9)
Disease status			
Cancer or diagnosed with tumor	NA	NA	17 (1.6)
Diabetes	NA	NA	86 (8.1)
Physical disability or bed bound	NA	NA	41 (3.9)
Chronic respiratory illness	NA	NA	139 (13.2)
Hypertension/heart disease	NA	NA	117 (11.1)
Musculoskeletal disorders	NA	NA	55 (5.2)
Multimorbidity	NA	NA	92 (8.7)
Kidney problems	NA	NA	11 (1.0)

Abbreviation: NA, not applicable.

The study sample included individuals with chronic conditions (558 [52.8%]) and those without (499 [47.2%]). Chronic diseases in the sample included cancer (17 participants [1.6%]), diabetes (86 participants [8.1%]), disability or stroke (41 participants [3.9%]), respiratory illness (139 participants [13.2%]), hypertension and/or heart disease (117 participants [11.1%]), musculoskeletal disorders (55 participants [5.2%]), kidney problems (11 participants [1.0%]), and multimorbidity (92 participants [8.7%]). Therefore, common chronic diseases among refugee populations included diabetes, hypertension, cardiovascular diseases, and chronic respiratory diseases.

### Overall QOL Among Adults With or Without Chronic Conditions

**Figure 1.**
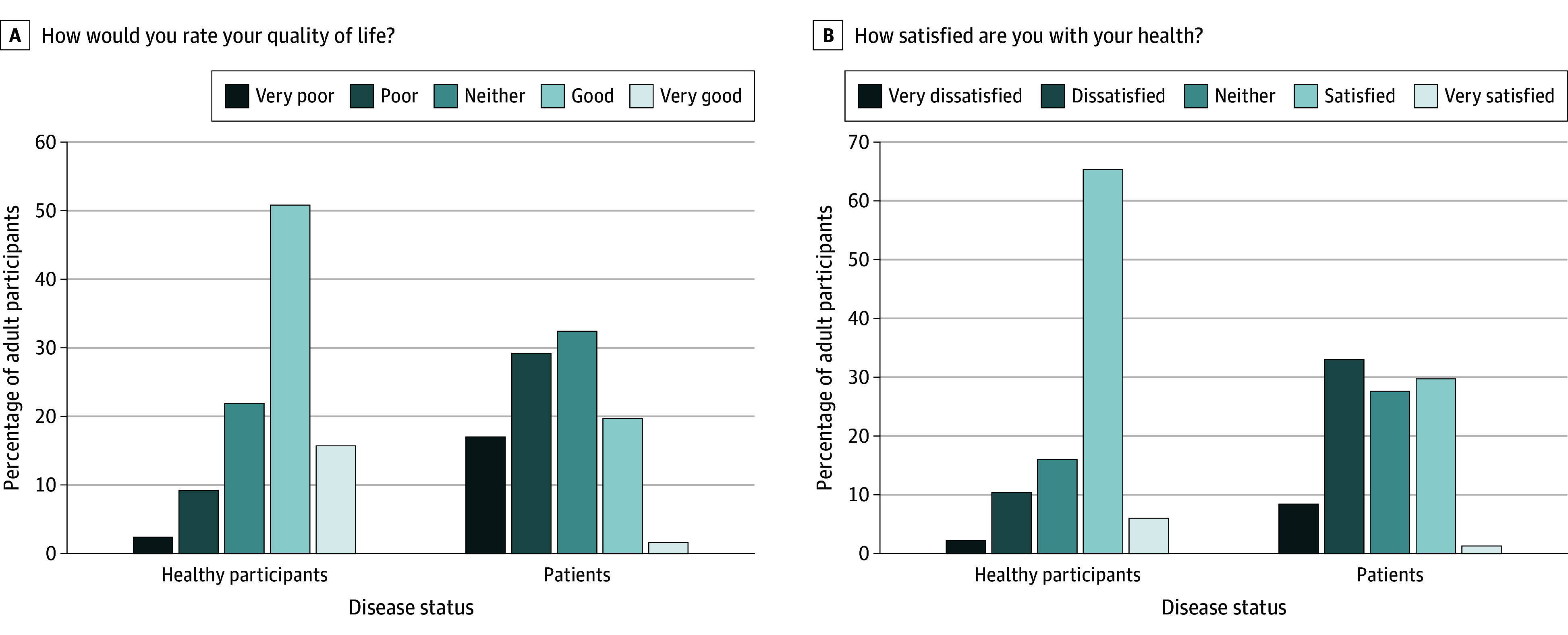
Overall Quality of Life Ratings and Overall Satisfaction With Health Ratings Among Rohingya Respondents

The first 2 questions of WHOQOL-BREF, related to overall QOL and overall health status, were assessed independently. Figure 1 depicts the overall QOL ratings among Rohingya respondents. Within healthy individuals, 332 (66.5%) rated their QOL as either good or very good, whereas 118 of the patients with chronic diseases (21.1%) provided similar ratings. Conversely, 260 of the participants with chronic diseases (46.6%) reported either very poor or poor QOL, while this was the case for 58 of the healthy individuals (11.6%). Figure 1 also illustrates overall satisfaction with health ratings among Rohingya respondents. Among healthy individuals, 63 (12.6%) provided ratings of either poor or very poor, while 231 of patients (41.4%) gave similar ratings regarding overall satisfaction with health.

### QOL Measures by Different Demographic Groups

In the study, we used 2-sample Wilcoxon tests and Kruskal-Wallis tests as statistical methods to investigate the associations between the domains of QOL and a range of sociodemographic factors. eFigure 1, eFigure 2, eFigure 3, and eFigure 4 in Supplement 1 show boxplots detailing the QOL scores based on demographic factors provided. Analysis of these scores indicated no significant difference in physical health between male and female participants. However, physical health scores tended to decrease with age. Additionally, individuals with chronic conditions demonstrated markedly lower physical health scores in contrast to healthy individuals. Furthermore, those without any formal education exhibited notably lower physical health scores compared with those with some level of schooling. Among employment status groups, individuals who were not working displayed the lowest scores for physical health.

The analysis in the psychological health, social relationships, and environmental domains revealed a pattern akin to the one observed in the physical health domain concerning demographic factors. Factors such as age, disease status, schooling, and employment status were found to be significantly associated with psychological health, social relationship, or environmental domains.

### Domains of QOL Among Adults With or Without Chronic Conditions

**Figure 2.**
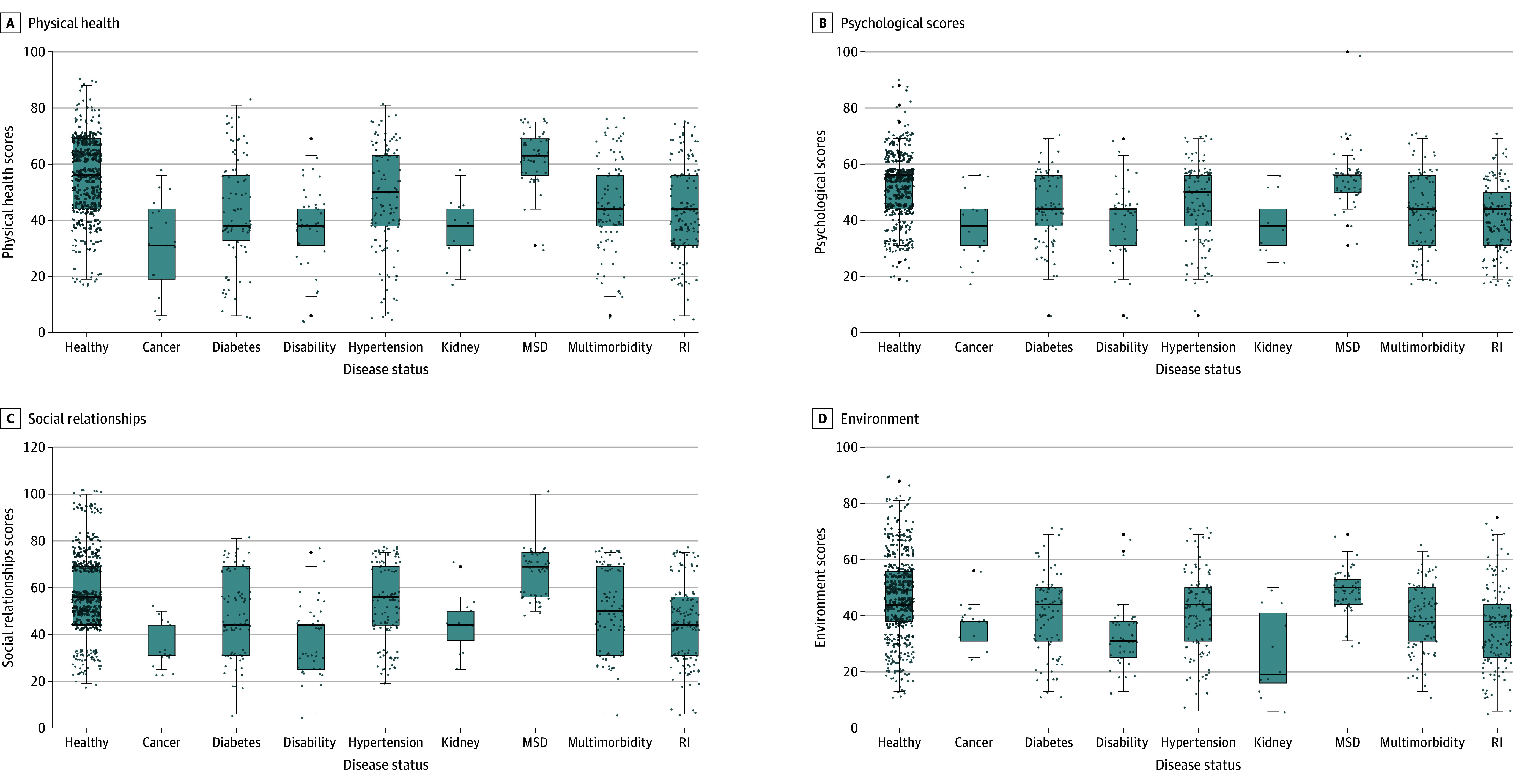
Rohingya Respondents' Quality of Life Across Different Disease Statuses Center line indicates median, box edges indicate IQRs, and dots indicate quality of life scores. MSD indicates musculoskeletal disorder; RI, chronic respiratory illness.

The study assessed QOL across different disease statuses, visualized in Figure 2. Healthy individuals demonstrated the highest scores across the 4 domains. Despite being healthy, their median scores of about 50 out of 100 suggested relatively poor QOL among Rohingya refugees. Among those with specific diseases, patients with cancer reported the poorest QOL across all domains. For instance, in the physical health domain, patients with cancer had a median (IQR) score of 29.8 (19.1-41.8), and patients with chronic kidney disease had a median (IQR) score of 35.1 (31.5-41.7). Patients with hypertension and those with musculoskeletal disorders had slightly higher QOL scores, with medians over 50. Participants with multimorbid conditions or respiratory illness reported similar patterns in their QOL across the 4 domains. The study used Tobit regression to analyze the QOL scores concerning disease status, and results are shown in Table 2, showing a decrease in all QOL domains across various diseases. Specifically, patients with cancer demonstrated the poorest QOL scores in every domain assessed. The findings derived from Tobit regression revealed that individuals with different health conditions exhibited lower QOL across all assessed domains in comparison with healthy individuals living in the Rohingya camps.

**Table 2.** The Tobit Regression for Domain Scores of Quality of Life Across Different Disease Status^a^

Chronic illness	Quality of life domains							
	Physical		Psychological		Social relationships		Environmental	
	β	*P* value	β	*P* value	β	*P* value	β	*P* value
Cancer/tumor	−3.703^b^	<.001	−2.213^b^	<.001	−3.83^b^2	<.001	−1.510	<.001
Diabetes	−2.590^b^	<.001	−1.614	<.001	−1.855	<.001	−1.366	<.001
Disability or had stroke or bed-bound	−2.760^b^	<.001	−1.820	<.001	−3.135^b^	<.001	−2.321^b^	<.001
Kidney problems	−2.144	<.001	−1.390	<.001	−2.096	<.001	−1.569	<.001
Hypertension/heart disease	−2.30	<.001	−1.556	<.001	−1.518	<.001	−1.256	<.001
Musculoskeletal disorders	−0.549	.004	−0.372	.01	−0.472	.12	−1.012	<.001
Multimorbidity	−3.394^b^	<.001	−2.484^b^	<.001	−2.787^b^	<.001	−2.301^b^	<.001
Chronic respiratory Illness	−1.756	<.001	−1.508	<.001	−1.735	<.001	−1.555	<.001

^a^ Reference category is healthy participants.

^b^ Indicates the maximum decreases of quality score by the disease.

### Domains of QOL by Demographic Factors: Tobit Regression

**Figure 3.**
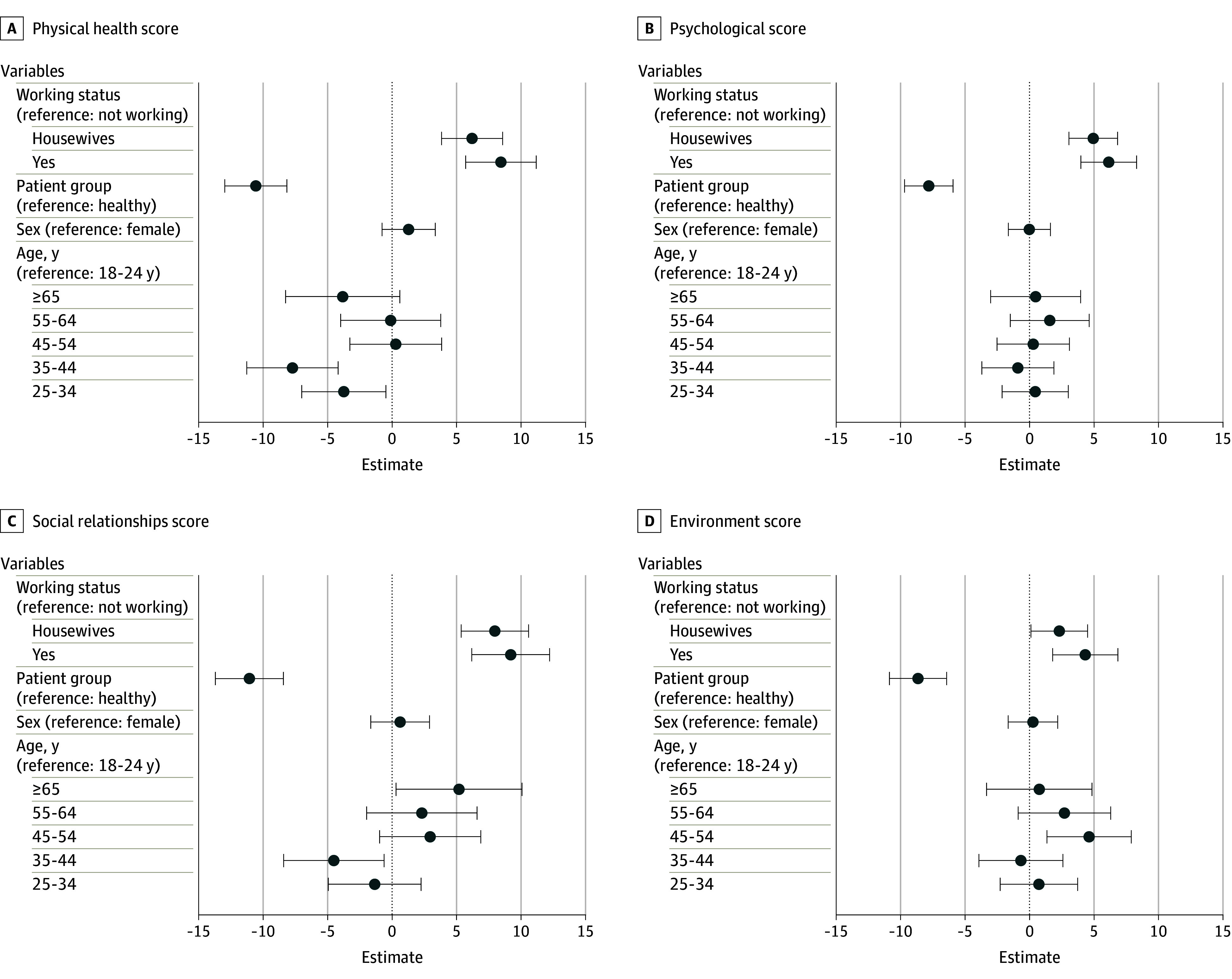
Coefficient of Tobit Regression Corresponding to Demographic Factors

We used Tobit regression with QOL scores and demographic factors. The results are presented in Figure 3. The results of the Tobit regression analysis show a statistically significant decline in both physical health scores (10.57 decrease; 95% CI, −12.97 to −8.17) and psychological domain scores (7.20 decrease; 95% CI, −9.71 to −5.93) among the patient group in comparison with the healthy individuals. Moreover, working individuals showed higher scores in both the physical health and psychological domains of QOL, with an increase of 8.45 and 6.15, respectively, compared with the nonworking group. Additionally, housewives exhibited better scores in the physical health (an increase of 6.20) and psychological (an increase of 4.95) domains compared with the nonworking group.

## Discussion

In the refugee camps of Cox’s Bazar, Bangladesh, over 1 million Rohingya people reside, with over 742 000 having lived there since 2017.^1^ Despite some improvements, refugees still have cramped shelters, inadequate access to resources, and uncertain futures. These challenging conditions, compounded by past traumas, may contribute to poor QOL. This cross-sectional study comprehensively explores the QOL experienced by resettled Rohingya refugees, analyzing sociodemographic factors, chronic illnesses, and living conditions within the camps.

The study reveals a significant disparity in the QOL between healthy individuals and those experiencing chronic illnesses among the Rohingya refugees. Specifically, 46.6% of participants with chronic diseases reported poor or very poor QOL compared with only 11.6% of healthy individuals. This stark difference highlights the severe outcomes chronic illnesses have on refugees’ well-being. In various refugee communities globally, a varying proportion of health-related QOL has been observed. Although the scale for health-related QOL is different than ours, Greece asylum seekers observed a similar pattern of QOL.^26^ Another study conducted with Filipino people who used drugs found the QOL ratings ranged between 12.6 and 14.1 out of a total score of 20, which is approximately equivalent to between 63 and 70 on the WHOQOL-BREF.^27^ Chronic diseases pose a major challenge for refugee health and well-being in camp settings. Moreover, refugees with chronic illnesses face significant barriers in accessing necessary health care, further diminishing their QOL.

This study finds chronic illnesses like cancer and multimorbid conditions were significantly associated with poor QOL among Rohingya refugees. Individuals grappling with chronic illnesses may experience physical limitations, pain, and emotional distress, all of which can negatively influence their perceived QOL.^28,29^ A few studies found that Rohingya refugees with chronic diseases are at risk of experiencing poor long-term physical health outcomes and reduced QOL.^30,31,32,33,34^ Refugees with cancer face significant difficulties accessing necessary care, as cancer treatment may be considered too complex, expensive, or inaccessible. We also found that individuals with disabilities in the Rohingya refugee camps had a poor QOL. A previous study determined that female refugees with disabilities are at greater risk of experiencing poor mental health outcomes.^3^ Lack of sufficient and appropriate resources and services has been a concern for refugees with disabilities, who have low QOL when accessing care at facilities lacking an inclusive environment.^3,31^

Within the physical health and psychological domain of this study, Rohingya refugees with cancer, multimorbidity, and disabilities exhibit the lowest QOL compared with their healthier counterparts. A few studies noted that refugees experiencing high levels of psychological distress also demonstrate poor QOL.^3,31,35^ The persistent shortage of mental health clinicians and services in the camps has been an ongoing concern for Rohingya refugees.^3,13^ In other contexts, long-term psychotherapy has been beneficial in enhancing the QOL of severely traumatized refugees with chronic conditions. Access to adequate health care, medication, and rehabilitation services becomes pivotal in mitigating the impact of chronic illnesses on daily life. Living conditions in refugee camps profoundly impact the QOL for Rohingya refugees.^13^ Overcrowded shelters, insufficient food, limited clean water, and inadequate sanitation create a challenging environment, hampering daily activities and posing health risks.^13,19^

We found the lowest QOL in the environmental domain among Rohingya with disabilities and multiple morbidities. Similarly, Syrian refugees experienced poor health-related QOL due to living conditions.^36^ The uncertain future and prolonged displacement add psychological stressors, worsening overall well-being. Although social networks in camps might support the social domain of QOL, they do not protect patients with chronic diseases or improve their QOL.

In the study, sociodemographic factors significantly shaped the QOL for Rohingya refugees, including age, education, and paid work opportunities. Older individuals had additional health and mobility challenges, affecting their overall well-being. Unlike other populations, sex dynamics in the Rohingya community do not influence access to resources and opportunities, so they do not affect the QOL differently for men and women.^13^ Limited educational opportunities hinder socioeconomic advancement, impacting overall QOL. Similar factors affect Afghan refugees in Greece and Syrian refugees in Sweden.^36,37^ Creating employment opportunities for refugees can significantly enhance their overall QOL. Offering paid employment addresses multiple dimensions of well-being by providing a sense of purpose, financial stability, and opportunities for growth, and it can also reduce the burden of mental health symptoms.

In this study, working individuals had better QOL scores in both physical and psychological domains compared with nonworkers. Housewives also had better QOL scores than those not working, suggesting that employment and even unpaid work benefit both physical and mental health. Employment provides a sense of purpose, social interaction, and financial resources, all of which enhance QOL. Similarly, housework offers a sense of purpose and social support from family members. This finding aligns with other studies on the Rohingya population regarding mental health symptoms.^3,19^

The study highlights the multifaceted challenges faced by the Rohingya refugees in Cox’s Bazar and highlights the critical need for comprehensive interventions to improve their QOL. Addressing health disparities, improving living conditions, providing mental health support, and creating opportunities for employment and education are essential steps toward enhancing the well-being of this vulnerable population. The findings serve as a call to action for policy makers, humanitarian organizations, and the international community to prioritize the needs of the Rohingya refugees and implement sustainable solutions to improve their QOL.

### Limitations

While the study provides valuable insights into the QOL among Rohingya refugees in Bangladesh, it is important to acknowledge certain limitations that may impact the interpretation and generalizability of the findings. The study’s sample may not fully represent the diversity of the Rohingya refugee population. Those with severe health conditions, limited mobility, or language barriers may be underrepresented, potentially skewing the results. The cross-sectional nature of the study limits the ability to establish causation or understand the temporal associations between variables. Longitudinal studies would provide a more comprehensive understanding of changes in QOL over time. The reliance on self-reported data on QOL may introduce the potential for recall bias and social desirability bias. Participants may provide responses they believe are socially acceptable rather than reflecting their true experiences. The study may not fully capture the intricacies of the Rohingya culture and the specific challenges faced by the population.

## Conclusions

The study underscores the persistent challenges Rohingya refugees from Myanmar face in achieving a satisfactory QOL, even after 5 years of resettlement in Bangladesh. This emphasizes the critical need for a comprehensive health care program and strategy aimed at enhancing the 4 key dimensions of QOL: physical, psychological, social, and environmental. Notably, individuals with chronic conditions, including musculoskeletal disorders, cancer, and multimorbid conditions, exhibited heightened and compounded vulnerability, leading to an overall poor QOL. This highlights the disproportionate burden of chronic illnesses among the Rohingya refugee population. Addressing the QOL of Rohingya refugees in Bangladesh requires a holistic approach that considers sociodemographic factors, the impact of chronic illnesses, and the enhancement of living conditions within the refugee camps.

## Notes

Supplement 1. **eFigure 1. **The QOL Across Physical Health Domain Score **eFigure 2.** The QOL Across Psychological Domain Score **eFigure 3. **The QOL Across Social Relationship Domain Score **eFigure 4. **The QOL Across Environmental Domain Score **eTable 1.** Sample Allocation of Patients From Kutupalong Rohingya Camp **eTable 2. **Sample Allocation of Healthy Individuals From Kutupalong Rohingya Camp **eAppendix.** Calculation of Response Rate **eTable 3.** Proportion of Missing Data in Covariates

Supplement 2.
Data Sharing Statement
