# Error in Author Information

**DOI:** 10.1001/jamanetworkopen.2025.10491

**Published:** 2025-04-14

In the Original Investigation titled “Chronic Illness and Quality of Life 5 Years After Displacement Among Rohingya Refugees in Bangladesh,”^1^ published September 17, 2024, there was an error in the author information. Juwel Rana’s degree should have been listed as MPH. This article has been corrected.^1^
