# Supplementary material for: Chronic Illness and Quality of Life 5 Years After Displacement Among Rohingya Refugees in Bangladesh
Source: JAMA Netw Open. 2024 Sep 17;7(9):e2433809. doi: 10.1001/jamanetworkopen.2024.33809 (PMC11409150; doi:10.1001/jamanetworkopen.2024.33809)
Supplement: Supplement 1. — eFigure 1. The QOL Across Physical Health Domain Score eFigure 2. The QOL Across Psychological Domain Score eFigure 3. The QOL Across Social Relationship Domain Score eFigure 4. The QOL Across Environmental Domain Score eTable 1. Sample Allocation of Patients From Kutupalong Rohingya Camp eTable 2. Sample Allocation of Healthy Individuals From Kutupalong Rohingya Camp eAppendix. Calculation of Response Rate eTable 3. Proportion of Missing Data in Covariates [file jamanetwopen-e2433809-s001.pdf]

Supplemental Online Content

Hossain A, Baten RBA, Sadif A, et al. Chronic illness and quality of life after displacement among Rohingya refugees in Bangladesh. *JAMA Netw Open*. 2024;7(9):e2433809. doi:10.1001/jamanetworkopen.2024.33809

eFigure 1. The QOL Across Physical Health Domain Score
eFigure 2. The QOL Across Psychological Domain Score
eFigure 3. The QOL Across Social Relationship Domain Score
eFigure 4. The QOL Across Environmental Domain Score
eTable 1. Sample Allocation of Patients From Kutupalong Rohingya Camp
eTable 2. Sample Allocation of Healthy Individuals From Kutupalong Rohingya Camp
eAppendix. Calculation of Response Rate
eTable 3. Proportion of Missing Data in Covariates

This supplemental material has been provided by the authors to give readers additional information about their work.

© 2024 Hossan A et al. *JAMA Network Open.*

eFigure 1. The QOL Across Physical Health Domain Score

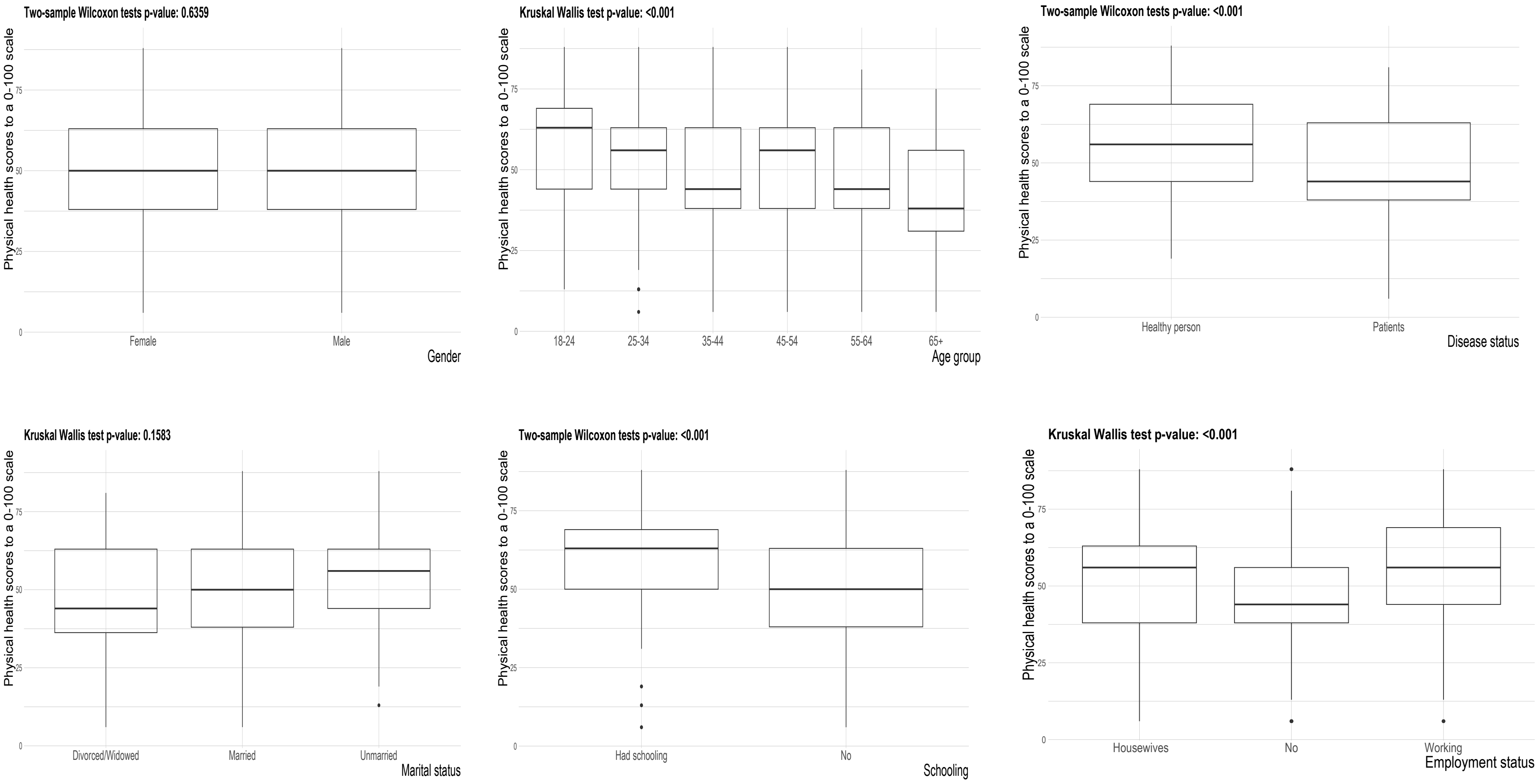

© 2024 Hossan A et al. *JAMA Network Open.*

eFigure 2. The QOL Across Psychological Domain Score

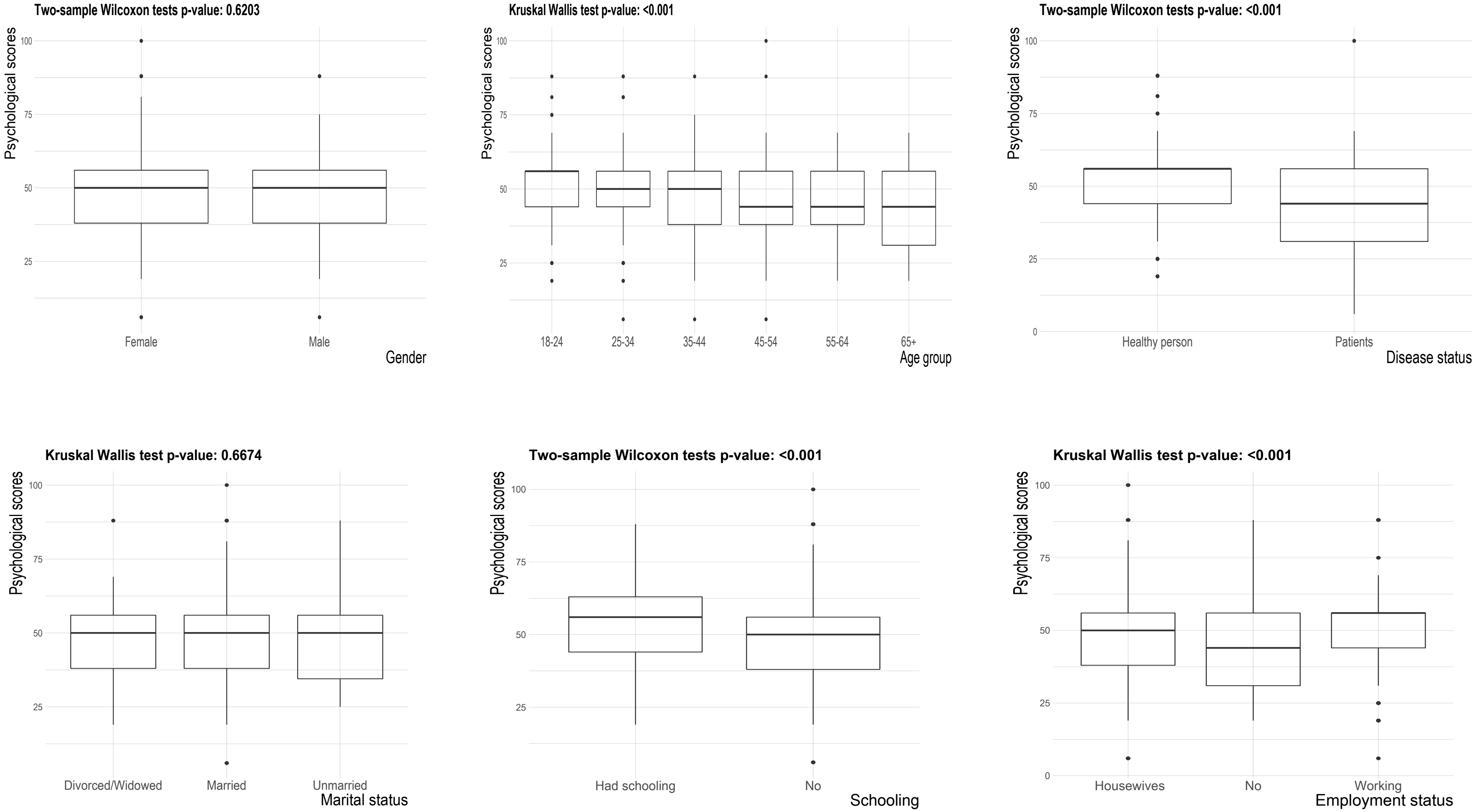

© 2024 Hossan A et al. *JAMA Network Open.*

eFigure 3. The QOL Across Social Relationship Domain Score

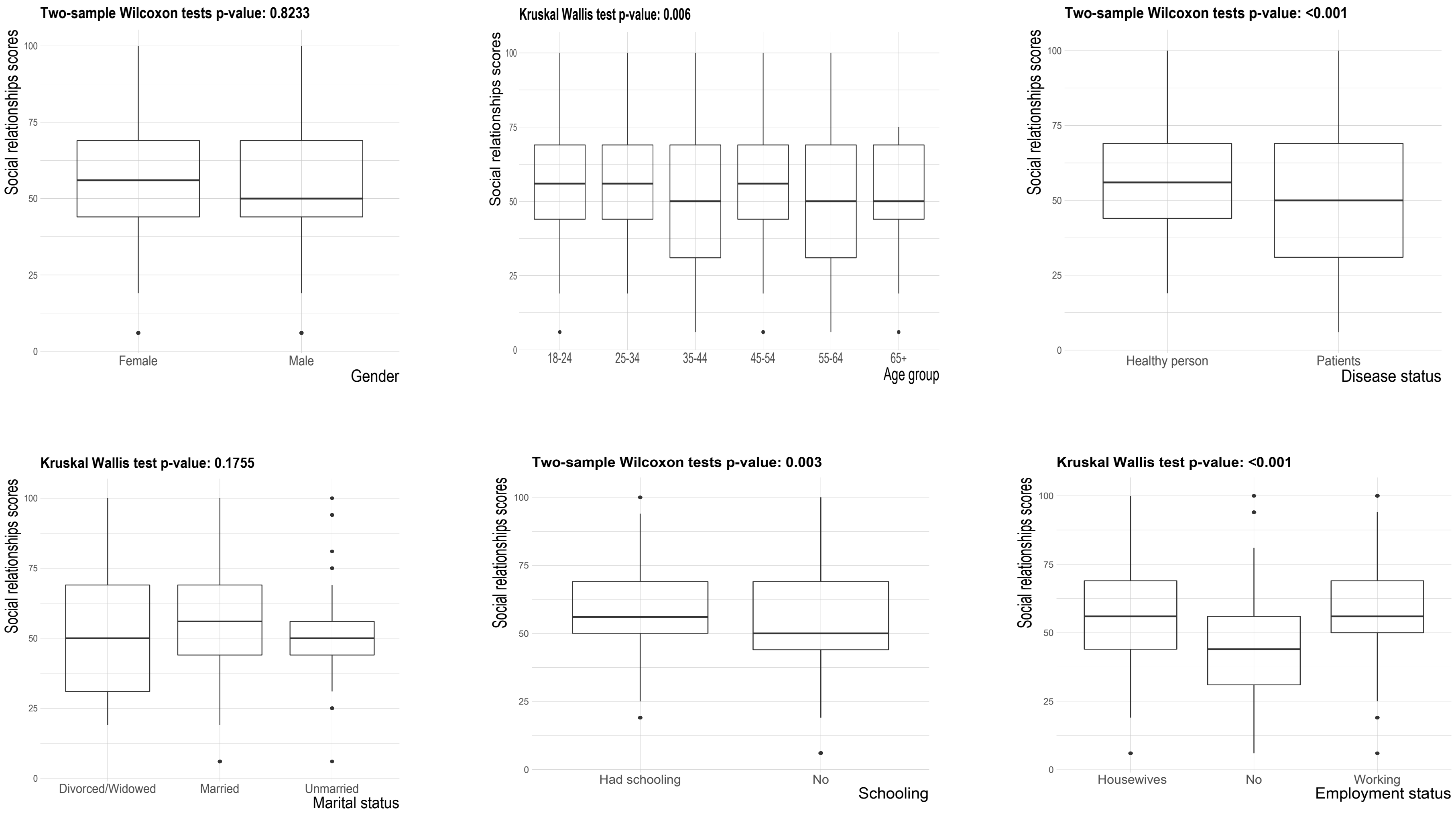

© 2024 Hossan A et al. *JAMA Network Open.*

eFigure 4. The QOL Across Environmental Domain Score

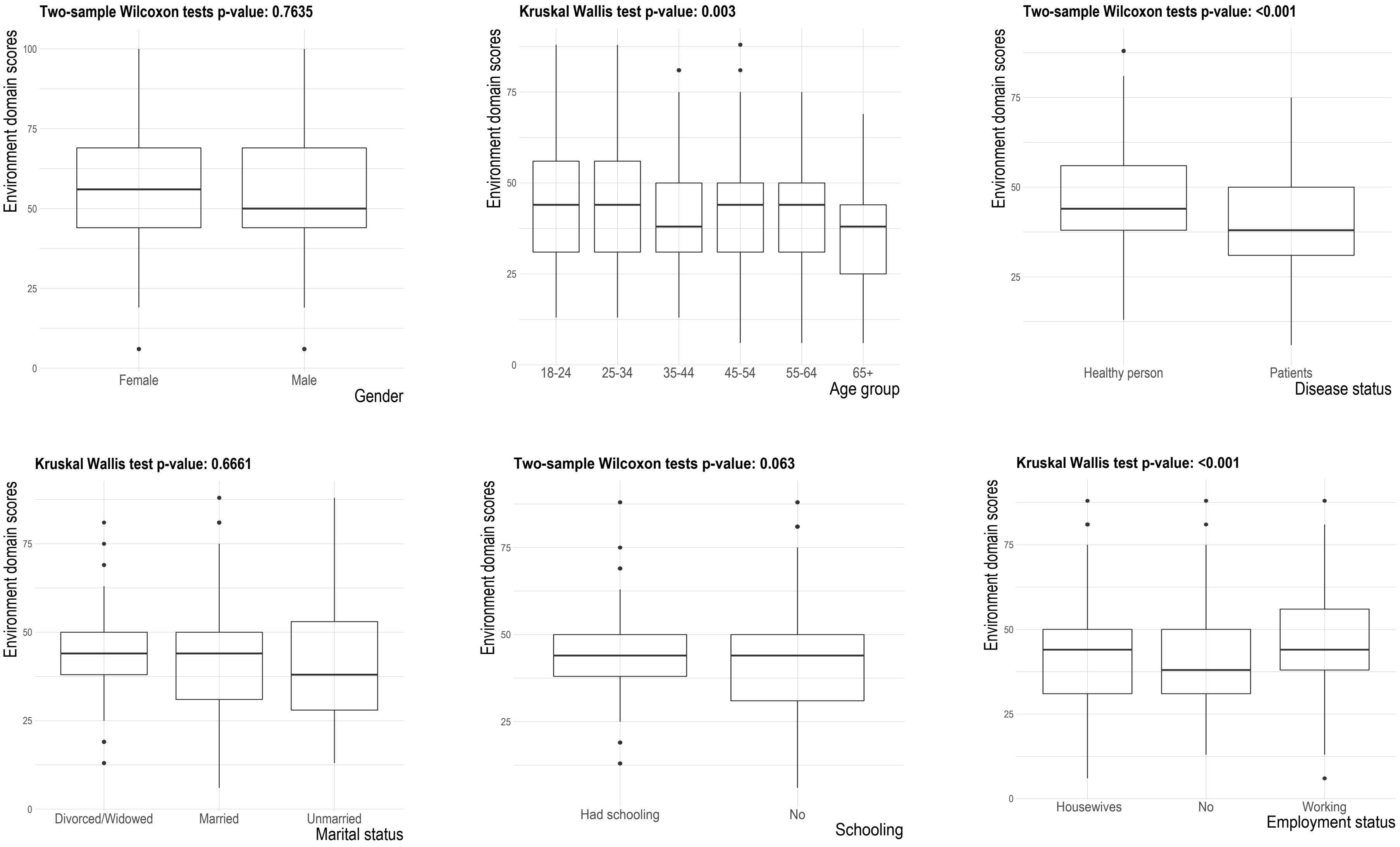

© 2024 Hossan A et al. *JAMA Network Open.*

eTable 1. Sample Allocation of Patients From Kutupalong Rohingya Camp

Organization	Medical centre	Camps name	Sample size
HMBD	1	Camp 7	164
HMBD	2	Camp 4	148
YPSA	1	Camp 2W	93
YPSA	2	Camp 6	71
YPSA	3	Camp 5	88
		Total	564

eTable 2. Sample Allocation of Healthy Individuals From Kutupalong Rohingya Camp

Camps name	Households	Populaiton	Sample size
Camp 7	5458	44965	105
Camp 4	7191	28263	96
Camp 17	1127	1740	78
Camp 2W	5458	28095	84
Camp 6	5694	27144	80
Camp 5	6153	29789	76
		Total	519

Source of Households and population: Action Contre la Faim Oxfam Save the Children (2018) Rohingya Refugee Response Gender Analysis: Recognizing and responding to gender inequalities Available: https://reliefweb.int/sites/reliefweb.int/files/resources/rr-rohingya-refugee-response-gender-analysis-010818-en.pdf (Accessed on October 17, 2022)

© 2024 Hossan A et al. *JAMA Network Open.*

eAppendix. Calculation of Response Rate

The response rate (RR) is the number of complete interviews with reporting units divided by the number of eligible reporting units in the sample. The following formula was used to calculate the response rate [1].

$$RR1 = \frac{I}{(I + P) + (R + NC + O) + (UH + UO)}$$

Where, RR1 = Response rate
I = Complete interview
P = Partial interview
R = Refusal and break-off
NC = non-contact
O = Other
UH = Unknown if household/occupied HU
UO = Unknown, other

The details of these quantities for our study are given in the following:

I and P=The in-person household/clinic survey was conducted in which housing units/ clinic are sampled from an address-based sampling frame of eight selected camps using systematic sampling technique. We consider less than 50% of all applicable questions answered (with other than a refusal or no answer) equals break-off, 50%-80% equals partial, and more that 80% equals complete. We found complete answered from 1058 participants (i.e., I=1184). We found 1 of the healthy participants did not complete the questionnaire for QOL (i.e., P=1).

R= Refusals and break-offs consist of cases in which some contact has been made with the housing unit/ clinic and a responsible household member/ patient has declined to do the interview, or an initiated interview results in a terminal break-off. Seventeen of the respondents refused to take part in the interview, but no one took a terminal break-off after initiating an interview (i.e., R=17).

© 2024 Hossan A et al. *JAMA Network Open.*

NC= Non-contacts in in-person household/ clinic surveys consist of three types: a) unable to gain access to the building, b) no one reached at housing unit, and c) respondent away or unavailable. The housings/ clinic were accessible in the camps and we were able to reach at housing unit during the interview period (i.e., NC=0).

O= Other cases represent instances in which the respondent is/was eligible and did not refuse the interview, but no interview is obtainable because of: a) death; b) the respondent is physically and/or mentally unable to do an interview; c) language problems; and d) miscellaneous other reasons. We did not face any language problem to exclude participants. We found 8 physically ill (bed-bound or palliative care) patients from household during the interview (i.e., O=.

UH= Cases of unknown eligibility and no interview include situations in which it is not known if an eligible housing unit exists and those in which a housing unit exists, but it is unknown whether an eligible respondent is present in the housing unit. It was not a scenario in our situation (i.e., UH=0).

UO= Not eligible cases for in-person household surveys include: a) out-of-sample housing units; b) not-a-housing unit; c) vacant housing units; d) housing units with no eligible respondent; and e) situations in which quotas have been filled. In a systematic sampling technique, we found a total of 57 households without any adult respondents during the interview (UO=57).

Thus, the response rate for healthy individuals, RR=499/ (499+4+7+0+3+0+6) = 96.1% and the response rate for patient individuals, RR=558/ (558+4+7+0+3+0+0) = 98.0%

Reference:

[1] The American Association for Public Opinion Research. 2016. *Standard Definitions: Final Dispositions of Case Codes and Outcome Rates for Surveys. 9th edition*. AAPOR.

© 2024 Hossan A et al. *JAMA Network Open.*

eTable 3. Proportion of Missing Data in Covariates

**Variable (Available data)**	Missing (%)
**Gender (N= 1058)**	1 (0.17%)
**Age** (in years) (N= 1058)	1 (0.17%)
**Marital Status (N=1058)**	1 (0.17%)
**Education (N=1058)**	1 (2.36%)
**Paid-work status in the last month (N=1058)**	11 (1.60%)
**Disease status**	0 (0%)

© 2024 Hossan A et al. *JAMA Network Open.*
