# Supplementary material for: Chronic Illness and Quality of Life 5 Years After Displacement Among Rohingya Refugees in Bangladesh
Source: JAMA Netw Open. 2024 Sep 17;7(9):e2433809. doi: 10.1001/jamanetworkopen.2024.33809 (PMC11409150; doi:10.1001/jamanetworkopen.2024.33809)
Supplement: Supplement 2. — Data Sharing Statement [file jamanetwopen-e2433809-s002.pdf]

Data Sharing Statement

Hossain. Chronic Illness and Quality of Life Displacement Among Rohingya Refugees in Bangladesh. *JAMA Netw Open*. Published September 17, 2024.
doi:10.1001/jamanetworkopen.2024.33809

Data
Data available: Yes
Data types: Deidentified participant data
How to access data: The de-identified dataset, protocol, permission letter from the international rescue committee (IRC), and questionnaire can be found in the source https://osf.io/tymd9/.
When available: With publication

Supporting Documents
Document types: Statistical/analytic code
How to access documents: The de-identified dataset, protocol, permission letter from the International Rescue Committee (IRC), and questionnaire can be found in the source https://osf.io/tymd9/.
When available: With publication

Additional Information
Who can access the data: Ahmed Hossain
Types of analyses: For a specified purpose
Mechanisms of data availability: With investigator support
Any additional restrictions: NA
